# Modeling the chemical dynamics of chloride ion indicators

**DOI:** 10.1186/1471-2202-13-S1-P113

**Published:** 2012-07-16

**Authors:** Alexander J Redford, Susan Ingram, Alexander Dimitrov

**Affiliations:** 1 Department of Mathematics, Washington State University, Vancouver WA 98686, USA; 2 Department of Neurological Surgery, Oregon Health & Science University, Portland OR 97239, USA

## 

Fluorescent indicators have shown promise as a relatively non-invasive probe to measure cytosolic ion concentrations in brain slices and neuron cultures using microscopy imaging. Most fluorescent ion indicators bind selectively with a certain ion in solution causing a decrease in fluorescence in a process known as quenching. Under steady state conditions, a fluorescence measurement, made at a specific point and time, is directly related to the local ion concentration at the same point and time, typically via the Stern-Volmer relationship. However, this is usually no longer true under the dynamic conditions inside a cell when transmembrane currents are active.

In our research, we are interested in measuring chloride (Cl^-^) channel currents because of their implication in substance abuse mechanisms. Useful probes have been found in Cl^-^ sensitive dyes, such as *N*-(6-methoxyquinolyl) acetoethyl ester (MQAE) [1], and 6-methoxy-*N*-ethylquinolinium (MEQ) [3], and variants of the Yellow Fluorescent Protein (YFP) useful as a Cl^-^ biosensor in dendritic compartments [4].

We have modeled jointly the Cl^-^ flow as part of a Hodgkin-Huxley type model and the interaction of Cl^-^ with a generic binding indicator as a system of non-linear differential equations derived from biophysical and chemical kinetic theory, using arbitrary parameters. Using the quenched indicator concentration as the given *input signal*, several approximate solutions can be derived for the corresponding *output signal*, the Cl^-^ current of interest. To date, we have explored a simple quasi-linear approximation similar to that discussed in [2], as well as a best-fit model-based approximation in which the output is assumed to satisfy a sum of parameterized exponential curves (an assumption consistent with the theory of neuron excitation). We consider how both approximations can be optimally applied to a noisy input signal, and on their limitations in probing disparate time scales and small currents. We also test these methods on current experimental data obtained as part of study of the biophysics of drug addiction (Ingram et. al, *in progress*).

**Figure 1:**
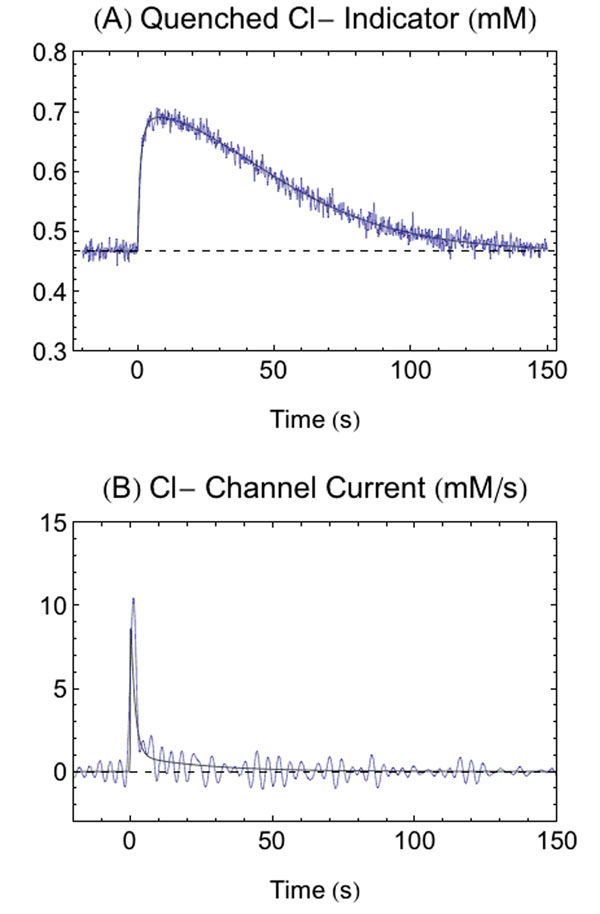
Joint model of Cl- channel current and Cl- indicator binding. A) Concentration of quenched indicator due to current in B, with simulated noise. B) Cl- channel current reconstructed from noisy model (blue) and actual channel current (black).
